# Supplementary material for: Changing health compliance through message repetition based on the extended parallel process model in the COVID-19 pandemic
Source: PeerJ. 2020 Nov 2;8:e10318. doi: 10.7717/peerj.10318 (PMC7643556; doi:10.7717/peerj.10318)
Supplement: Supplemental Information 1 [file peerj-08-10318-s001.pdf]

Supplementary 1

**Preliminary Experiment**

The preliminary experiment was conducted to 1) decide what model to examine in the main experiment, and 2) test the validity of the items that will be used in the main experiment.

**Method**

The method is the same as that described in the manuscript, except for the sample size.

**Results**

We used the *lavaan* package in R to perform structural equation modeling (SEM) based on data from 372 users of the Yahoo! Crowdsourcing Service in Japan. There were no missing data. We chose a robust maximum likelihood estimator, which produces Satorra-Bentler rescaled $\chi^2$ to correct for non-normality-induced bias. Table 1' shows the correlation table with means and standard deviations, and the theoretical model is presented in Figure 1' (standardized parameter estimates provided). The comparative fit index (CFI) = .972, Tucker-Lewis index (TLI) = .963, and root mean square error of approximation (RMSEA) = .041. These values indicate a good fit between the model and the observed data. We also performed validity tests for items 1–12, and present the results in Table 2'. The results indicate that the items used in the study are valid.

**Table 1'. Correlations for SEM analyses**

| Observed variable | 1 | 2 | 3 | 4 | 5 | 6 | 7 | 8 | 9 | 10 | 11 | 12 | 13 | 14 |
|---|---|---|---|---|---|---|---|---|---|---|---|---|---|---|
| 1. item 1 | 1 | | | | | | | | | | | | | |
| 2. item 2 | 0.583 | 1 | | | | | | | | | | | | |
| 3. item 3 | 0.698 | 0.664 | 1 | | | | | | | | | | | |
| 4. item 4 | 0.425 | 0.455 | 0.447 | 1 | | | | | | | | | | |
| 5. item 5 | 0.490 | 0.500 | 0.534 | 0.731 | 1 | | | | | | | | | |
| 6. item 6 | 0.372 | 0.384 | 0.371 | 0.717 | 0.673 | 1 | | | | | | | | |
| 7. item 7 | 0.143 | 0.117 | 0.154 | 0.057 | 0.159 | 0.080 | 1 | | | | | | | |
| 8. item 8 | 0.176 | 0.132 | 0.127 | 0.045 | 0.188 | 0.085 | 0.754 | 1 | | | | | | |
| 9. item 9 | 0.080 | 0.148 | 0.112 | 0.065 | 0.133 | 0.081 | 0.482 | 0.523 | 1 | | | | | |
| 10. item 10 | 0.189 | 0.223 | 0.236 | 0.169 | 0.300 | 0.176 | 0.386 | 0.362 | 0.341 | 1 | | | | |
| 11. item 11 | 0.137 | 0.168 | 0.206 | 0.191 | 0.272 | 0.202 | 0.180 | 0.219 | 0.214 | 0.628 | 1 | | | |
| 12. item 12 | 0.116 | 0.184 | 0.172 | 0.173 | 0.245 | 0.140 | 0.316 | 0.278 | 0.304 | 0.799 | 0.680 | 1 | | |
| 13. Behavior Intention | 0.478 | 0.474 | 0.597 | 0.452 | 0.520 | 0.409 | 0.142 | 0.131 | 0.139 | 0.169 | 0.204 | 0.150 | 1 | |
| 14. Repetition | 0.145 | 0.011 | 0.139 | 0.074 | 0.062 | 0.074 | -0.071 | -0.055 | -0.146 | -0.004 | -0.027 | 0.008 | 0.187 | 1 |

Note. The variables were standardized to have a mean of 0 and a standard deviation of 1. $N = 372$.

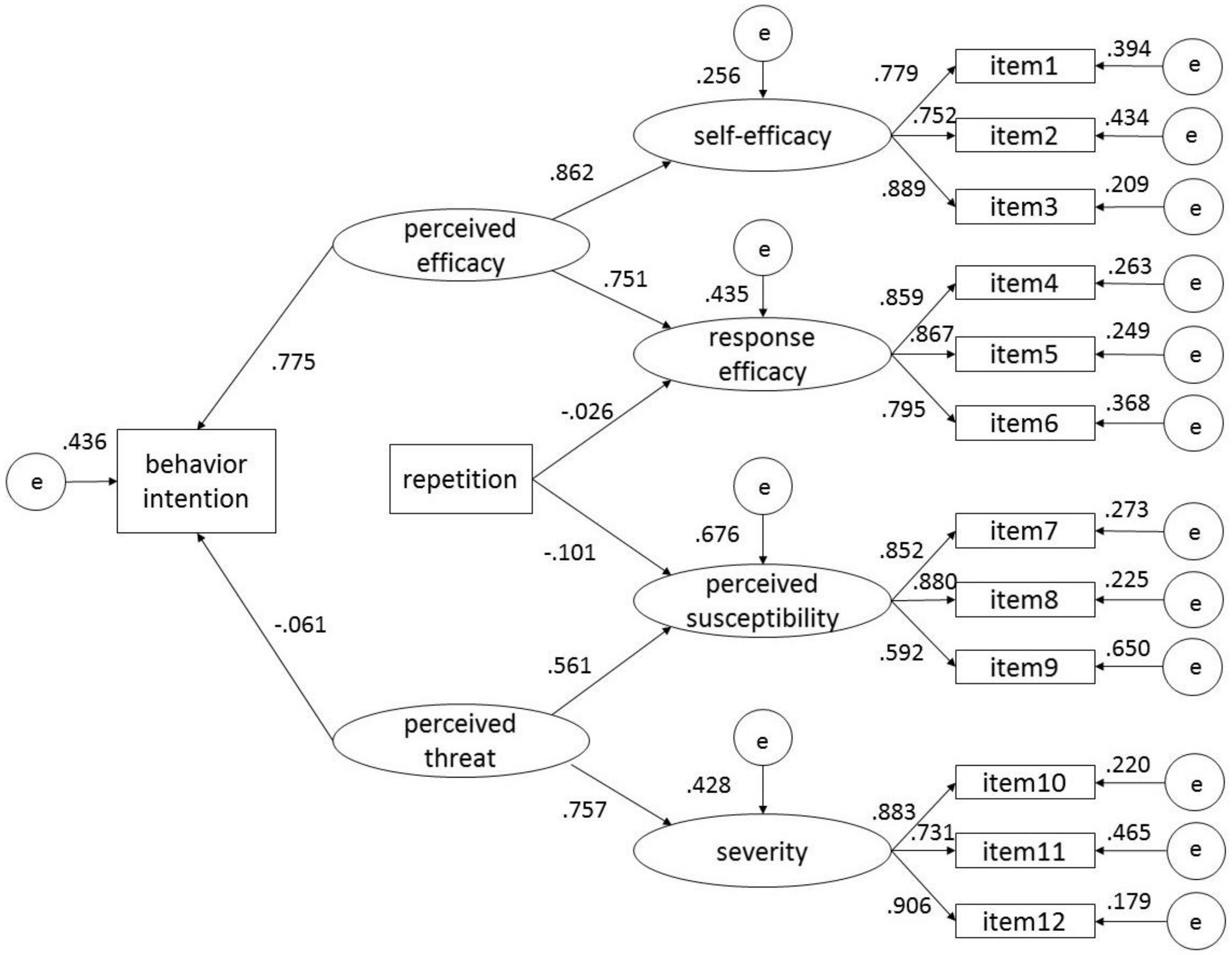


**Figure 1’. The theoretical structure equation model and its results**

CFI = .972; TLI = .963; RMSEA = .041; degrees of freedom = 70; e = error.

**Table 2’. Validity test for items 1–12**

| | self-efficacy | response efficacy | perceived susceptibility | severity |
|---|---|---|---|---|
| average variance extracted (AVE) | 0.654 | 0.707 | 0.671 | 0.712 |
| square root of AVE | 0.809 | 0.841 | 0.785 | 0.844 |

convergent validity: AVE > .5

**Inter-construct correlations**

| | self-efficacy | response efficacy | perceived susceptibility | severity |
|---|---|---|---|---|
| self-efficacy | 1 | | | |
| response efficacy | 0.420 | 1 | | |
| perceived susceptibility | 0.043 | 0.033 | 1 | |
| severity | 0.077 | 0.059 | 0.018 | 1 |

discriminant validity: square root of AVE > inter-construct correlations

## References


Curran, P. J., West, S. G., & Finch, J. F. (1996). The robustness of test statistics to nonnormality and specification error in confirmatory factor analysis. *Psychological Methods*, *1*(1), 16.

Finney, S. J., & DiStefano, C. (2006). Non-normal and categorical data in structural equation modeling. *Structural Equation Modeling: A Second Course*, *10*(6), 269-314.

Hair, J., Black, W., Babin, B., & Anderson, R. (2010). *Multivariate data analysis* (7th ed.): Prentice-Hall, Inc. Upper Saddle River, NJ, USA.

Hu, L. T., & Bentler, P. M. (1999). Cutoff criteria for fit indexes in covariance structure analysis: Conventional criteria versus new alternatives. *Structural Equation Modeling: A Multidisciplinary Journal*, *6*(1), 1-55.

Rosseel Y (2012). "lavaan: An R Package for Structural Equation Modeling." *Journal of Statistical Software*, *48*(2), 1-36. http://www.jstatsoft.org/v48/i02/.

Satorra, A., & Bentler, P. M. (1994). Corrections to test statistics and standard errors in covariance structure analysis. In A. von Eye & C. C. Clogg (Eds.), *Analysis of latent variables in developmental research* (pp. 399- 419). Newbury Park, CA: Sage.
